# Tracking COVID-19 using taste and smell loss Google searches is not a reliable strategy

**DOI:** 10.1038/s41598-020-77316-3

**Published:** 2020-11-25

**Authors:** Kim Asseo, Fabrizio Fierro, Yuli Slavutsky, Johannes Frasnelli, Masha Y. Niv

**Affiliations:** 1 The Institute of Biochemistry, Food Science and Nutrition, The Faculty of Agriculture, Food and Environment, The Hebrew University of Jerusalem, Rehovot, Israel; 2 Department of Statistics and Data Science, The Hebrew University of Jerusalem, Rehovot, Israel; 3 Department of Anatomy, University of Québec in Trois-Rivières, Trois-Rivières, QC Canada

**Keywords:** Public health, Epidemiology, Health care

## Abstract

Web search tools are widely used by the general public to obtain health-related information, and analysis of search data is often suggested for public health monitoring. We analyzed popularity of searches related to smell loss and taste loss, recently listed as symptoms of COVID-19. Searches on sight loss and hearing loss, which are not considered as COVID-19 symptoms, were used as control. Google Trends results per region in Italy or state in the US were compared to COVID-19 incidence in the corresponding geographical areas. The COVID-19 incidence did not correlate with searches for non-symptoms, but in some weeks had high correlation with taste and smell loss searches, which also correlated with each other. Correlation of the sensory symptoms with new COVID-19 cases for each country as a whole was high at some time points, but decreased (Italy) or dramatically fluctuated over time (US). Smell loss searches correlated with the incidence of media reports in the US. Our results show that popularity of symptom searches is not reliable for pandemic monitoring. Awareness of this limitation is important during the COVID-19 pandemic, which continues to spread and to exhibit new clinical manifestations, and for potential future health threats.

## Introduction

SARS-CoV-2 pandemic has by now hit almost all countries worldwide. Monitoring disease occurrence is a key prerequisite for combating its spread. The availability of laboratory tests differs from country to country, and many nations are not able to test the general and even the symptomatic population broadly.

Furthermore, symptoms elicited by SARS-CoV-2 are still being discovered, with the list of officially recognized symptoms being updated on a rolling basis. Smell loss (anosmia), and to a lesser degree taste loss (ageusia), accompanying COVID-19 infection have appeared in reports of COVID-19 patients’ testimonies^1^, preprints of scientific papers^2–5^, discussions by journalists, and now in numerous peer-reviewed publications (e.g.^6–11^). “New taste and smell loss” were added to fever, cough, shortness of breath or difficulty breathing, chill, muscle pain, headache, and sore throat, to the CDC-listed symptoms. The National Health Services of the UK describes “loss or change to your sense of smell or taste” among the symptoms and the World Health Organization has listed “loss of taste or smell” among “less common symptoms” of COVID-19. COVID-19-related taste and smell loss is in fact very common, but reported prevalence depends on the assessment method^12,13^.

Here we set out to explore the hypothesis, proposed by several groups^14–16^ and also discussed in The New York Times^17^ and on CNBC^18^, that Google Trends searches on smell loss are indicative of new COVID-19 cases.

We analyzed searches on smell loss, as well as those on taste loss. We used sight loss and hearing loss as controls, since these senses are not currently known to be impaired in COVID-19 patients. Because smell and taste loss are gradually becoming recognized as COVID-19 symptoms, we also looked at searches for ‘COVID-19 symptoms’ in general.

Based on previous reports^14–18^, we expected to find a correlation between the popularity of taste loss and smell loss searches and the number of new COVID-19 cases. We set out to check whether the level of correlation is conserved over time. Specifically, we assumed that media coverage may potentially decouple the search popularity from the number of cases, since searches would result not only from self-symptoms, but also from interest elicited by media coverage. Additionally, based on their co-occurrence in COVID-19 patients^4,11^, we hypothesized there should be a significant positive correlation between “taste loss” and “smell loss” searches if these are COVID-19 related.

Media reports on smell and taste loss as COVID-19 symptoms were analyzed using Media Cloud, a database collecting digital media articles (hence, not including radio, television or printed media) and quantifying the attention over time to a query topic.

We focused on two countries, US and Italy, where the incidence of new cases was high, but the dynamics over time, as well as media coverage, were different.

## Methods

### New cases

The number of new cases of COVID-19 for each of the 20 Italian regions was obtained from the Italian Ministry of Health website (https://www.salute.gov.it/portale/nuovocoronavirus/homeNuovoCoronavirus.jsp?lingua=english), and for the 51 US states from the Johns Hopkins Coronavirus Resource Center (https://coronavirus.jhu.edu/us-map).

The data for each individual region was normalized with respect to its total population. The number of inhabitants for each Italian region was retrieved from the last data available on the Istituto Nazionale di Statistica (National Institute for Statistics) website (https://dati.istat.it/Index.aspx?lang=en&SubSessionId=1d073136-f11a-4329-a1ed-3a3920a1ec32).

The population numbers for the US states were adapted from^19^.

### Search data

The search terms described in Table 1 were used as input in Google Trends (https://www.google.com/trends) and the number of searches for each region or state was collected. We were interested in terms representing only self-symptoms, (though the more generic option offered by Google Trends to search by topics provided similar results, not shown). Additional terms and different combinations of keywords were tested, resulting in no evident differences in the calculated correlations from the results obtained using the terms presented in Table 1, which we identified as the most popular ones. As an example, addition of personalized search terms such as “I can’t smell” or “I can’t taste” to the keywords listed in Table 1, did not change the obtained trend.

**Table 1:** Search terms used for searches in Italian and English on Google Trends. The first column is the reference keyword used to summarize the corresponding searches in the main text. The “ +” character allows one to sum up data for multiple searches with a logical OR.

	Italian keywords	English keywords
Taste loss	Perdita gusto + perdita del gusto	Taste loss + loss of taste
Smell loss	Perdita olfatto + perdita dell’olfatto	Smell loss + loss of smell
Sight loss	Perdita vista + perdita della vista	Vision loss + sight loss
Hearing loss	Perdita udito + perdita dell’udito	Hearing loss + loss of hearing
COVID symptoms	Sintomi coronavirus + sintomi COVID19 + sintomi COVID + coronavirus sintomi + COVID sintomi	COVID symptoms + coronavirus symptoms + COVID-19 symptoms + COVID19 symptoms + Coronavirus symptoms

Google Trends provides the normalized number of searches according to the population living in the country’s sub-area and assigns a popularity index to the keyword searches that spans a range from 0 to 100. A value of 100 is assigned to the region in which the keyword reaches the maximum volume of searches for the dates and countries selected, with no relation to the other keywords searched in that comparison.

In addition to the regional data, Google Trends popularity index of the taste loss and smell loss queries from March 4th to August 25th was calculated on a daily basis for each country as a whole. As for the Google Trends searches for regions/states, the data is automatically normalized by the webserver, assigning the day with the highest volume of searches the value of 100; other days were assigned values relative to that peak day.

### Correlations over regions/states

Pearson correlation between the number of new COVID-19 cases per 1,000,000 inhabitants and the search data for different symptoms, was calculated for each week separately, for four nonconsecutive weeks, representing different stages of the pandemic spread. Each observation is a region (Italy) or state (US). The *p* values of each correlation were adjusted using a false discovery rate^20^ adaptation for the different presented comparisons (3 in Italy and 5 in the US). Confidence intervals were calculated using Fisher transformation^21^ as described in^22^. The adjusted confidence levels were approximated according to the adjusted *p* values (see Supplementary Text S1).

To make sure that the correlation was not dominated by an outlier in the data, a test was done on the US data by removing New York—the state with the highest number of new COVID-19 cases during the initial weeks of analysis—and recalculating the correlations from the new data. The change in correlation was found insignificant (not shown).

### Sliding windows correlation (per country as a whole)

To estimate the correlation between search terms and the total number of new cases per country as a whole, a sliding window analysis was performed: after preliminary analysis with various window sizes, Pearson correlations (where the observations are the different days) were calculated separately for time frames of one month (31 days), shifted by one day at a time, resulting in a total of 172 windows. Since multiple comparisons were performed in each window, the *p* values of each estimate of the correlation were adjusted using a false discovery rate^20^.

Conservative confidence intervals which assume independence of the observations were calculated using Fisher transformation^21^ as described in the section “correlation over regions”.

### Media impact data

We used the Media Cloud webserver (https://www.mediacloud.org) to obtain an estimate for the number of times a certain keyword appeared on digital news on a daily basis in the period between March 4th to August 25th. By using the quoted version of the keywords as defined in Table 1 for taste loss and smell loss, the normalized numbers of appearances of these keywords in digital news were obtained. The collection of media used to search our keywords were the “Italy—National” and the “U.S. Top Sources 2018” available on the Media Cloud website.

In order to compare the Media Cloud results to the whole country Google Trends, Media Cloud data were normalized in the manner Google Trends are normalized: a value of 100 was assigned to the day with the highest media coverage peak of a particular search query, other days being assigned values relative to that day. The number of new cases shown in the same figure were normalized with respect to 1,000,000 inhabitants.

A sliding windows analysis was performed for the media/search popularity correlation calculation in the same way as described in the previous paragraph.

RStudio software^23^ was used to build all the graphs in the manuscript.

## Results

Does the number of searches for taste and smell loss in a region correspond to the number of new COVID-19 cases in that region? We analyzed Google searches and the numbers of new COVID-19 cases in Italy and the US, considering the states (US) and the regions (Italy) they are composed by. For each region or state, the following parameters were calculated: Google Trends popularity index per region/state for generic (‘COVID-19 symptoms’) and specific (‘taste loss’, ‘smell loss’) symptoms of COVID-19, as well as control keywords not known as COVID-19 symptoms (‘hearing loss’, ‘sight loss’); the number of new COVID-19 cases per region or state normalized per 1,000,000 inhabitants. Pearson correlation with the normalized number of new cases was calculated for each country and for different weeks.

**Figure 1:**
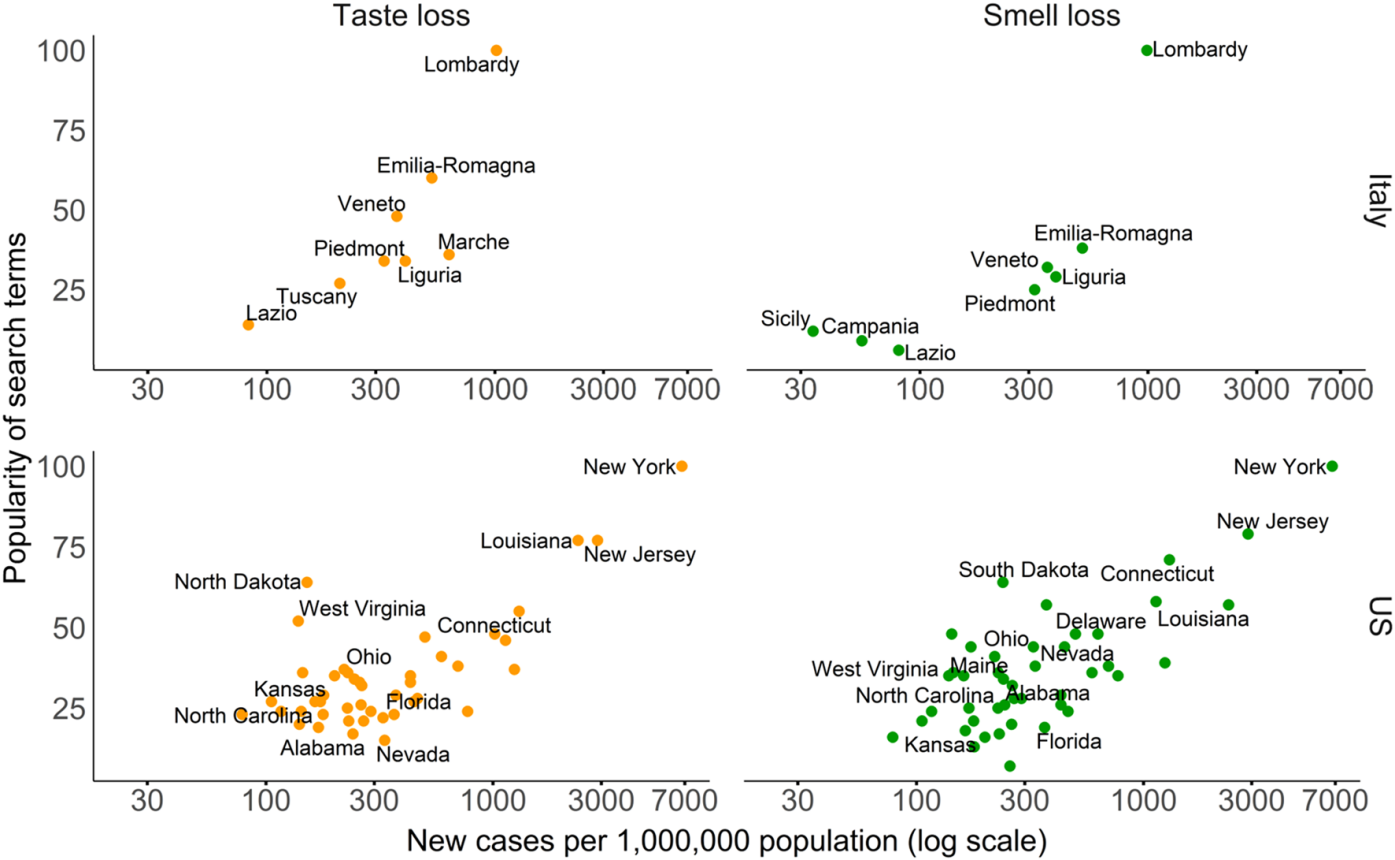
Data from which correlation was derived. Here shown for Italy (11–17 March) and the US (1–7 April), the weeks with highest correlation for taste loss and smell loss. The graphs show the taste loss (in orange) and smell loss (in green) search queries. Each point represents a different region/state. Normalized number of new cases related to the corresponding week on the x axis, popularity of the search terms for the same week on the y axis. For both Italy and the US graphs, not all the regions or states are shown because of the lack of popularity index for some regions/states.

Results for weeks representing a good correlation for taste loss and smell loss searches are shown in Fig. 1 (correlation of 0.91 (*p* = 0.007), 0.97 (*p* < 0.001) in Italy and 0.81 (*p* < 0.001) and 0.74 (*p* < 0.001) for the US for taste loss and smell loss respectively). The volume of searches for these two keywords was high, among the other regions, in Lombardy, Emilia Romagna and Veneto, as well as New York, New Jersey and Louisiana, which are geographical sub-areas with high rates of new COVID-19 patients/inhabitants in their respective country.

**Figure 2:**
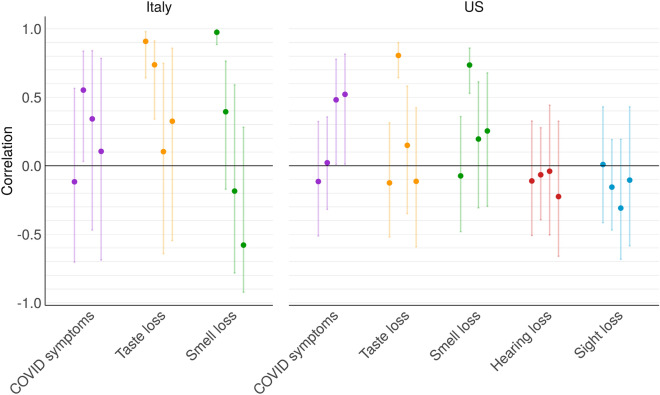
Correlation over geographical regions. For the weeks of 11–17 of March, 1–7 of April, 13–19 of May and 19–25 of August (in this order), correlations between the number of new COVID-19 cases (normalized with respect to 1,000,000 inhabitants) and popularity index of each search term are shown as round points. Confidence intervals (90%) calculated for each week separately are shown as error bars.

If the number of taste and smell loss searches is indeed indicative of new COVID-19 cases, such correlation should hold over time. In Fig. 2, we present data for four nonconsecutive weeks which span the period of March 4th till August 25th, 2020.

Overall, Fig. 2 illustrates that there were some weeks with strong correlations between the number of new cases and the number of searches for taste and smell loss, and relatively narrow confidence intervals (i.e. 11–17 of March in Italy and 1–7 of April in the US). This may be attributable to people affected by taste loss and/or smell loss searching for the specific symptoms they have experienced. However, in other weeks, the correlation is either low or insignificant and the confidence intervals for the correlations in the other respective weeks are very wide.

Conjunctivitis has been recently added by the WHO to the list of the less common COVID-19 symptoms, but it rarely interferes with eyesight^24^. Therefore, we used hearing and sight loss as control. In the US, correlation with these terms is low with wide confidence intervals, covering the middle range including zero. The corresponding searches for the Italian translation of the queries (“perdita udito” and “perdita vista”) did not produce enough results to show data relative to different regions and, consequently, also displayed no correlation. The correlation with the general search term for “COVID-19 symptoms” in both countries changes from week to week and has wide confidence intervals.

Thus, in summary—the correlation of COVID-19 incidence with popularity of taste loss, smell loss and COVID-19 symptoms fluctuates and has wide confidence intervals, while the correlation with popularity of the control search for hearing loss and vision loss is constantly low.

**Figure 3:**
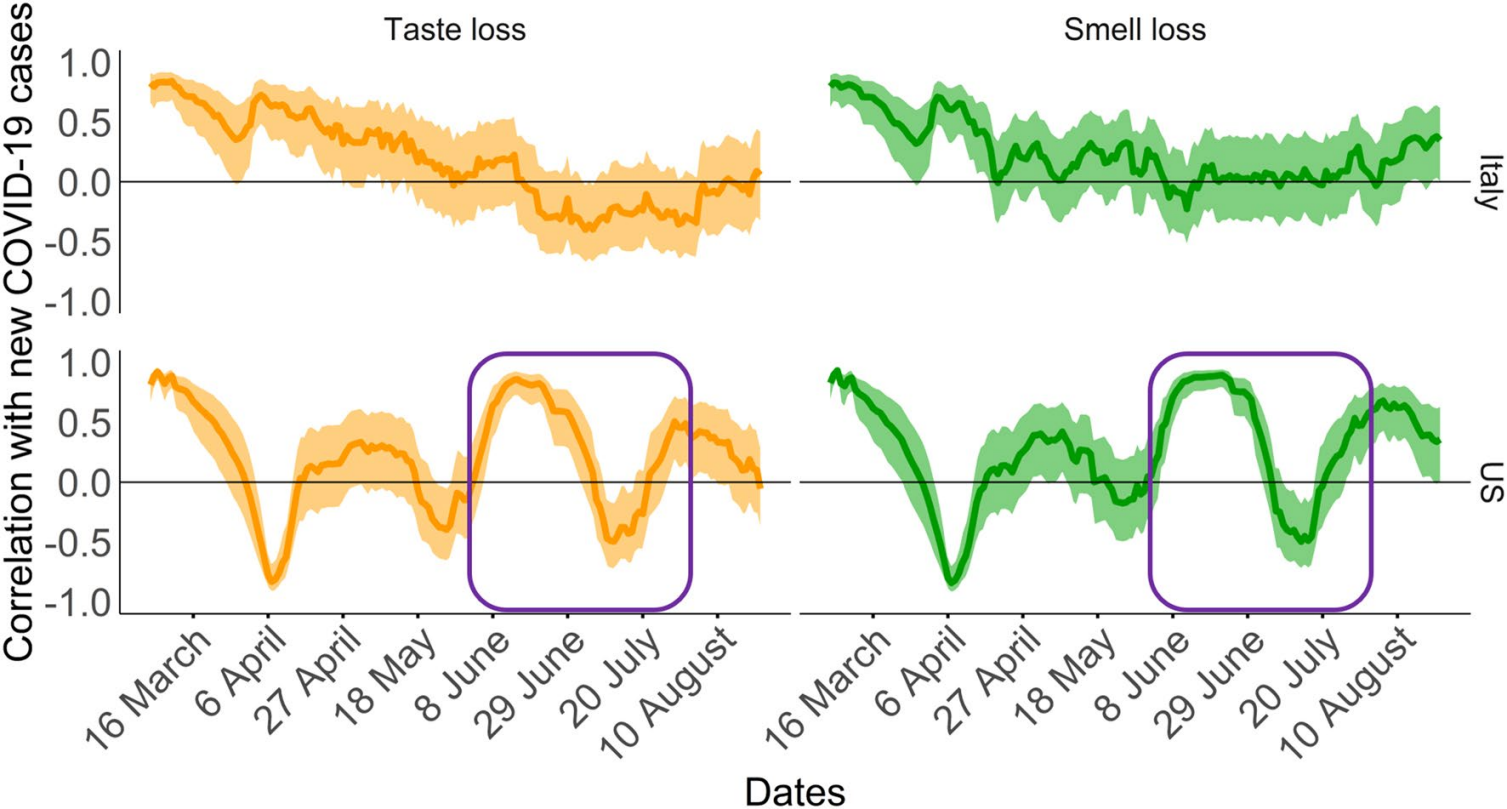
Sliding windows correlation data (time frame of 31 days). Correlation values between the total number of new COVID-19 cases in Italy and US, normalized with respect to 1,000,000 inhabitants, and search queries popularity index. Conservative confidence intervals of 90% are shown as ribbons. Correlation values including the dates in which a second increase in the number of new cases in the US is shown in a purple rectangle.

To better evaluate how the correlations evolve over time, the correlation between search terms popularity and total number of new cases per each country as a whole, was calculated for sliding windows (Fig. 3).

A gradual decrease in Italy and dramatic fluctuations in the US can be observed for the correlation between new cases and popularity of smell searches and of taste searches. While no strong second surge of new cases of COVID-19 occurred in Italy in the period under study, the US experienced a second increase in the number of new cases starting from the middle of June and reaching the highest peak on July 17th. During this period, the sliding windows correlation strongly fluctuates (purple rectangle in Fig. 3). This means that the number of Google searches is not at all representing the new cases incidence and is missing completely the dramatic and lasting increase in new cases.

We hypothesized that media coverage of COVID-19 related taste and smell symptoms may impact the number of searches. We therefore analyzed, on a daily basis and for each country as a whole, the number of new cases, as well as that of Google searches and the volume of digital media coverage of taste and smell loss.

**Figure 4:**
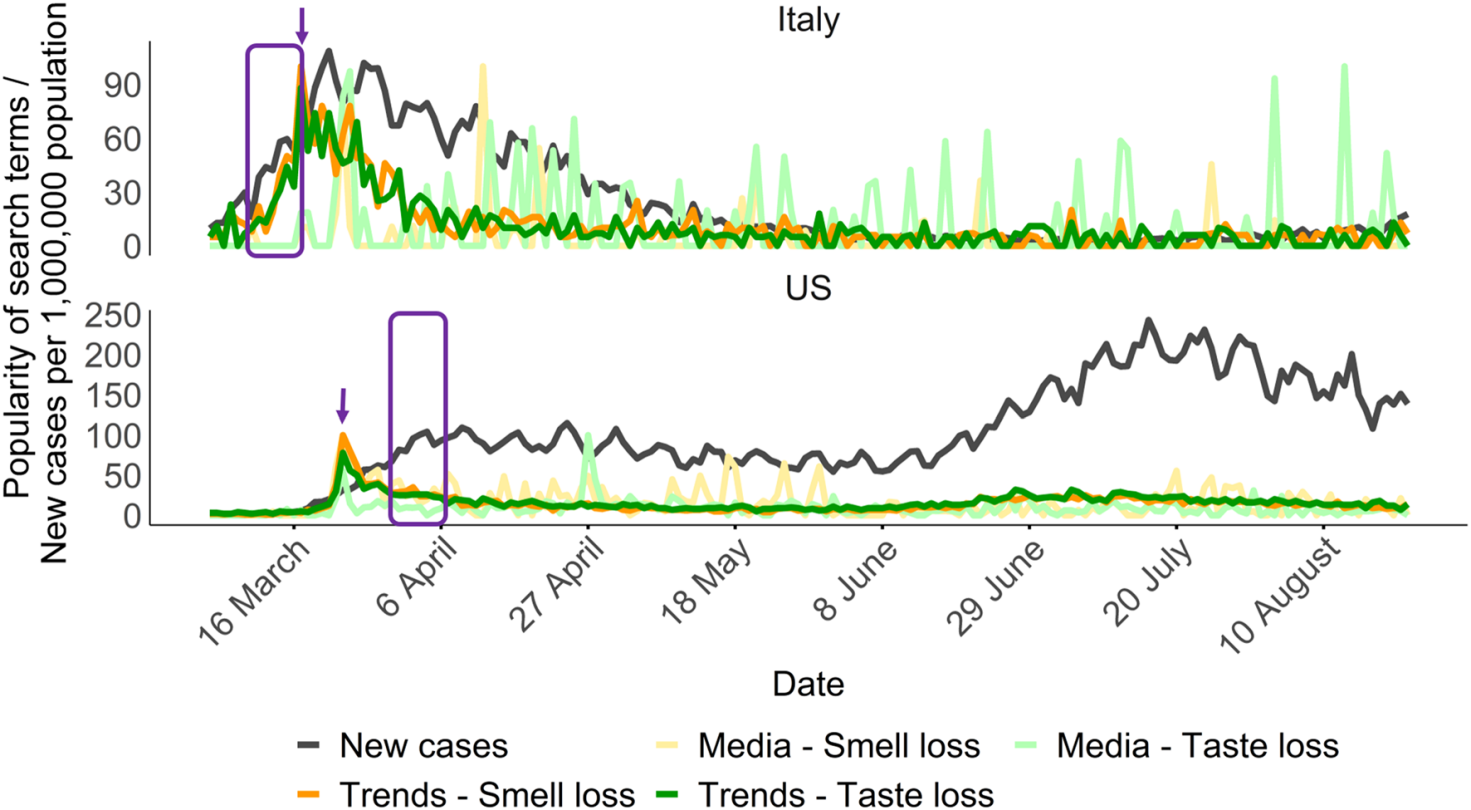
Media coverage. Comparison between Google searches volume for taste loss and smell loss queries, Media Cloud popularity of the same keywords and the number of new COVID-19 cases. Calculation was performed from March 4th to August 25th for Italy and the US. The Media Cloud data were normalized as for the Google search results, assigning the value of 100 to the day with the highest popularity, with the other days assigned values relative to that day. The number of new cases is relative to a population of 1,000,000 in the respective country. Purple rectangle highlights 11–17 March week in Italy, and 1–7 April week in the US, the arrows indicate the first peak for smell/taste loss media popularity.

Using the Explorer tool on the Media Cloud platform, we monitored the number of times taste loss and smell loss keywords were mentioned daily by digital news media, during the time period March 4th 2020–August 25th 2020 (Fig. 4).

The news about these two sensory-related symptoms, in both Italy and the US, were reported for the first time during the 11–17 March week, according to Media Cloud. Media coverage on the 4–10 March week in both Italy and the US actually reported the taste loss or smell loss in a different context, not related to COVID-19 symptoms. The first peak for media coverage in Italy was reached between the 23rd and 24th of March (purple arrow in the Italian graph of Fig. 4), while the correlation peak for Italy, reported in Figs. 1 and 2, was reached earlier, during the 11–17 March week (purple rectangle in the graph for Italy of Fig. 4). An increase in popularity of searches was observed before the first media reports of March 16th. Indeed, the total number of cases on March 16th in Italy was ~ 7 times higher than in the US (~ 30,000 for Italy vs ~ 4300 for the US) and the volume of searches observed up to that date was also higher in Italy (Supplementary Fig. S2).

The scenario we uncovered for the US was different. The week with the highest correlation is observed after the news became popular on digital media, differing from the Italian case (Fig. 4). The Media Cloud data and the Google Trends searches are perfectly superimposed in proximity of their first high peaks. The rise of cases in the US set in later than in Italy, and there was no time window in which the number of cases was surging while the taste and smell symptoms were still unknown. Indeed, the volume of searches in the US is more correlated with media trends (pair-wise correlation of 0.26 (*p* < 0.001), and 0.50 (*p* < 0.001) for taste and smell loss for the entire timeline) than in Italy (0.09 (*p* = 0.3), and 0.17 (*p* = 0.03)). The correlation between taste loss/smell loss searches popularity and Media Cloud data decreases and fluctuates over time for both countries (Supplementary Fig. S3).

Finally, smell and taste loss trends are closely related to each other (pair-wise correlation of 0.95 in the US and 0.89 in Italy over the entire time, *p* < 0.001).

## Discussion

In this study, we examined the correlation between Google searches for specific new symptoms of COVID-19 (taste loss and smell loss) and the number of people affected by the SARS-CoV-2 virus in Italy and the US. In general, on some weeks regions/states with a high percentage of infection cases tended to search for these specific symptoms more often than those with a low incidence when the number of new cases reaches a relatively high volume (~ 21,360 for Italy on 11–17 March and ~ 208,500 for the US on 1–7 April) for the first time. Several studies attempted to identify spikes in correlation between internet searches and number of COVID-19 cases^25–27^. However, our analysis showed that the correlation with searches for new symptoms dramatically varies over time, thus diminishing the initial appeal of this tool for monitoring the pandemic. We have also showed that even conservative calculations result in wide confidence intervals for these correlations. A striking example of lack of utility of Google Trends for monitoring COVID-19 spread is observed during the second surge of new cases in the US, when the number of infected patients keeps rising, while search popularity for smell or taste loss strongly fluctuates (Fig. 3 and Supplementary Fig. S4 for additional information).

Public perception of the pandemic may also alter the frequency of searches^28^. We found that media had an impact on the volume of searches, especially for smell loss in the US. In Italy the media effect on searches was milder, possibly due to the advanced state of the pandemic when the news started to appear on digital media. The Italian data likely suggests genuine interest based on self-symptoms even before they became broadly known to the general public. Fluctuations of the correlation curve over time is observed for both countries. Over the period studied here, the correlation between smell loss and media trends is higher than for taste, and both are higher for US than for Italy.

We found a strong correlation between searches for taste loss and for smell loss. This is in line with recent findings that the degree of COVID-19-related anosmia and ageusia correlate closely in affected individuals^4,11,29^, and with taste and smell being listed as a joint symptom by CDC (“new taste and smell loss”), NHS (“loss or change to your sense of smell or taste”) and WHO (“loss of taste or smell”).

As the pandemic continues to spread around the world, and new waves are expected, developing strategies relying on data retrieved from internet users' behavior for monitoring of COVID-19 hotspots remain in focus^30–33^. Due to the high popularity reached by the COVID-19 related topics, recently Google Trends has implemented the Coronavirus Search Trends tool to facilitate collection of virus related data (https://trends.google.com/trends/story/US_cu_4Rjdh3ABAABMHM_en). Google search trends have been already employed in the past for diseases monitoring, the most popular case represented by Google Flu Trends (https://www.google.org/flutrends/about/). Conflicting data regarding its reliability emerged from different studies, underlining the difficulties in the use of these methodologies for monitoring purposes^34,35^, and the service has been discontinued.

In conclusion, the results described here suggest that the correlation between searches of novel symptoms of an infectious disease and the number of new cases fluctuates and/or decreases over time. Relying solely on Google trends for taste loss and smell loss searches to monitor the spread of the SARS-CoV-2, as suggested by Goldman and Sachs^18^, is not a viable strategy. Nevertheless, utilization of information on smell and taste loss in sophisticated ways, that incorporate media coverage, saturation, and other effects, may be envisioned.

Limitations described here may apply also to new COVID-19 symptoms that are being discovered, such as skin lesions^36^, impairment of chemesthesis (a chemosensory modality that allows the perception of burning, cooling or tingling triggered by molecules)^11^, and more^37^.

Since future pandemics may, unfortunately, emerge^38^, it is fundamental to keep developing alternative monitoring strategies. The shortcomings of methods that rely on self-reporting should be kept in mind also in analyzing results from the various self-reporting apps^39–41^, and underscore the crucial role of independent epidemiological tools, such as sewage monitoring^42,43^ and widespread laboratory testing^33,44,45^.

## Supplementary information

Supplementary Information 1.

## Data Availability

The data that support the findings of this study are openly available in "GitHub" at https://github.com/KimAsseo/Google_COVID.
