# Supplementary material for: Tracking COVID-19 using taste and smell loss Google searches is not a reliable strategy
Source: Sci Rep. 2020 Nov 25;10:20527. doi: 10.1038/s41598-020-77316-3 (PMC7689487; doi:10.1038/s41598-020-77316-3)
Supplement: Supplementary file 1 — Supplementary Information 1. [file 41598_2020_77316_MOESM1_ESM.pdf]

Supplementary Information

Tracking COVID-19 using taste and smell loss Google searches is not a reliable strategy

Kim Asseo[1#], Fabrizio Fierro[1#], Yuli Slavutsky[2], Johannes Frasnelli[3] and Masha Y Niv[1*]

1 The Institute of Biochemistry, Food Science and Nutrition, The Robert H Smith Faculty of Agriculture, Food and Environment and The Fritz Haber Center for Molecular Dynamics, The Hebrew University of Jerusalem, Israel
2 Department of Statistics and Data Science, The Hebrew University of Jerusalem, Israel
3 Department of Anatomy, University of Québec in Trois-Rivières, Trois-Rivières, Québec, Canada
#equally contributing authors
**correspondence to masha.niv@mail.huji.ac.il*

Supplementary Text S1. For each comparison k (corresponding to the different symptoms) we denote the original p-values by $q_1^{(k)}, \ldots, q_n^{(k)}$ and the adjusted ones by $p_1^{(k)}, \ldots, p_n^{(k)}$ where $n = 4$ is the number of computed correlations, that is the four nonconsecutive weeks.

In order to achieve an overall confidence level of $1 - \alpha$, the adjusted confidence level $\tilde{\alpha}^{(k)}$ of each comparison was approximated by the maximal original p-value for which the adjusted one is smaller than α:

$$(1) \qquad \tilde{\alpha}^{(k)} = \max_i \left\{ q_i^{(k)} \middle| p_i^{(k)} < \alpha \right\}.$$

To achieve approximately normally distributed values, Fisher transformation was applied on the estimated correlations:

$$(2) \qquad z_r = \frac{1}{2} \ln \left(\frac{1+r}{1-r} \right).$$

Confidence intervals for the correlations $r_1^{(k)}, \ldots, r_n^{(k)}$ were obtained by computing

$$(3) \qquad z_l^{(k)}, z_u^{(k)} = z_r^{(k)} \pm z_{1-\frac{\tilde{\alpha}^{(k)}}{2}} \sqrt{\frac{1}{n-3}}$$

and the values z_l, z_u were transformed back to correlation scale using the inverse transformations:

$$(4) \qquad r_l = \frac{e^{2z_l^{(k)}} - 1}{e^{2z_l^{(k)}} + 1}$$

$$r_u = \frac{e^{2z_u^{(k)}} - 1}{e^{2z_u^{(k)}} + 1}.$$

Supplementary Figure S1 repeats the calculation presented in Figure 1 for a week with low correlation. This clearly illustrates the current lack of correlation between the number of new cases in each region (Italy) or state (US) and the popularity of Google searches on either taste or smell loss.

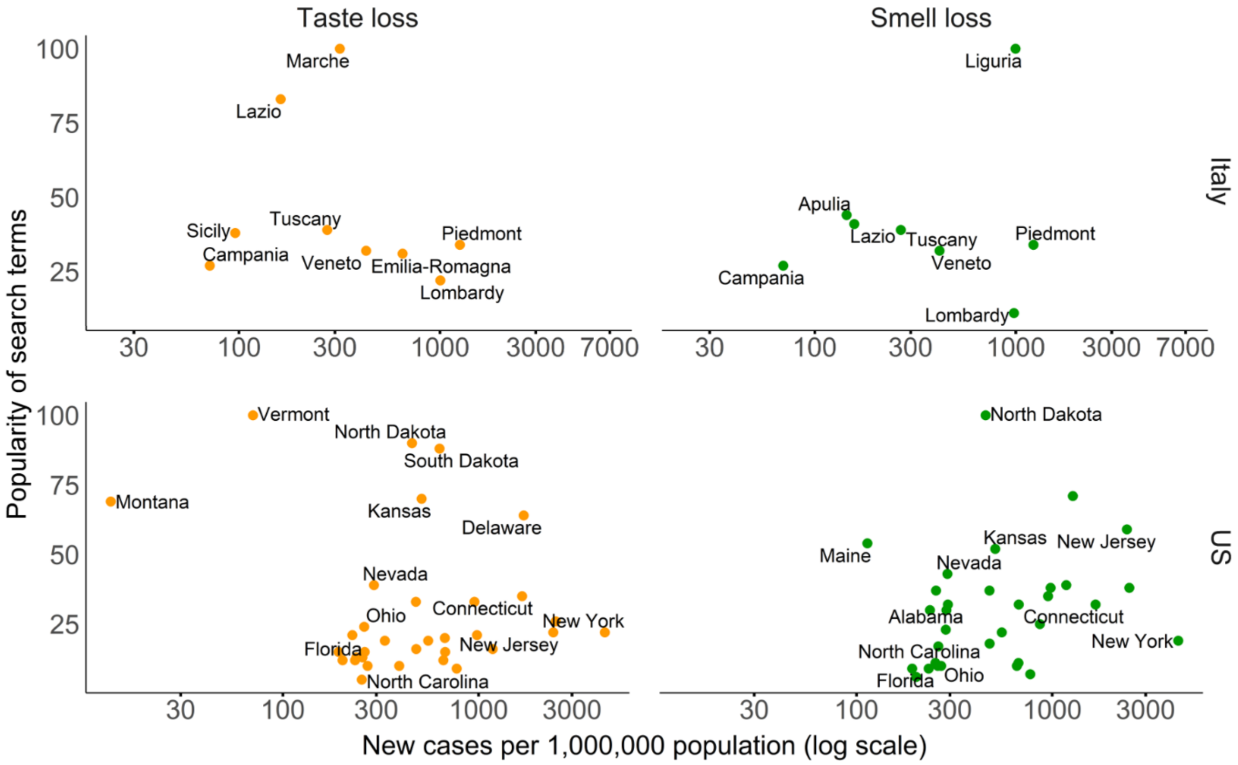

Figure S1: Correlation data for a low correlation week, 22-28 April 2020. The graphs describe the regions/states and include the taste loss (in orange) and smell loss (in green) search queries. Each point represents a different region/state (normalized number of new cases related to the corresponding week on the abscissa, popularity of the search terms on the ordinate axis). For both graphs, not all regions or states are shown because of the lack of popularity index for some of these geographical sub-areas.

Supplementary Figure S2 shows an increase in popularity of searches before the news appeared for Italy, but not for the US. It also exhibits a "surprise rise" in both countries with media coverage and new cases rising, followed by a "knowledge saturation", where the baseline of searches is somewhat higher than before, but overall low and not reflective of the number of new cases in the respective country.

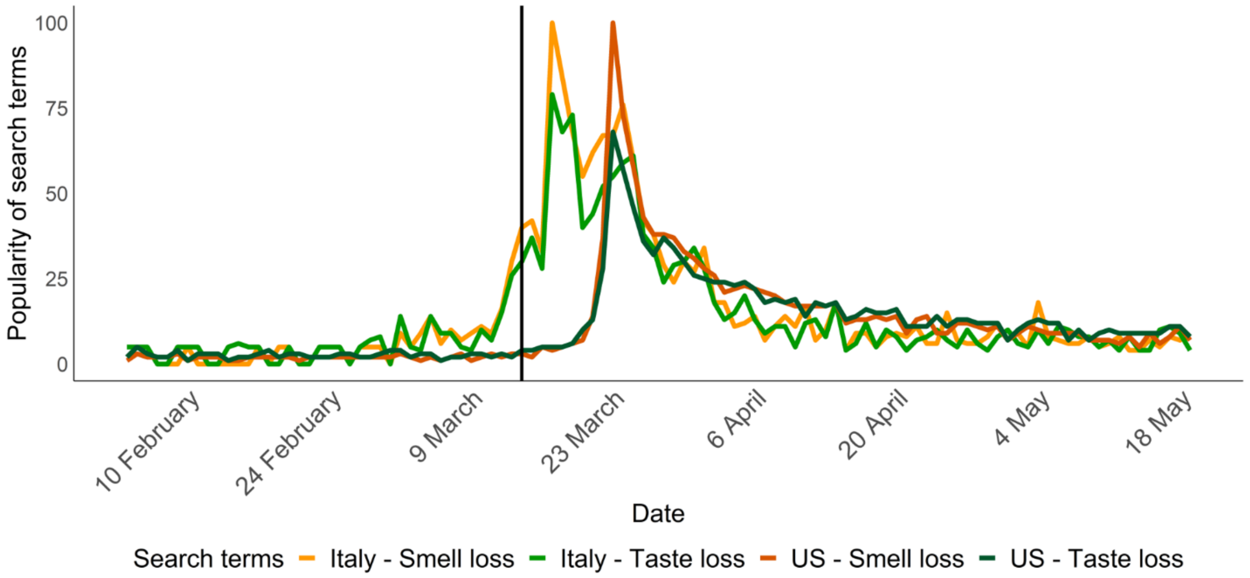

Figure S2: Popularity of smell loss and taste loss searches for Italy and the US from February 4th to May 19th. The former date lies one month before the point in time from which the records have been analyzed in the present study. The date the phenomenon was reported in the media for the first time is shown as a black vertical line.

Supplementary Figure S3 shows the correlation between Media Cloud popularity and search terms calculated using the sliding windows methodology. A high correlation is observed in March, especially in the US, when the news was initially reported by the media. Later on, the correlation fluctuates, never reaching the values of the first analyzed month, suggesting a decrease in interest due to the acquired concept by the population.

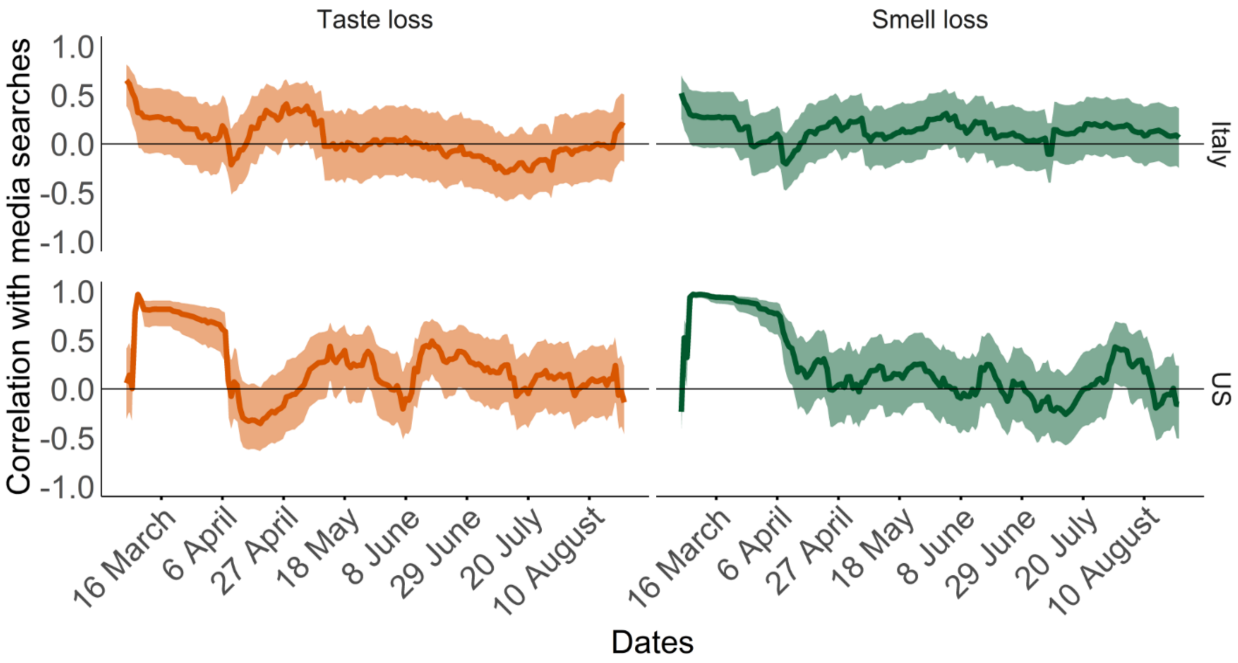

Figure S3: Sliding windows correlation for Media Cloud data (time frame of 31 days). Correlation between Media Cloud data and searches popularity calculated using the sliding windows methodology (see Methods). Confidence intervals of 90% are shown as ribbon.

As we submit the final version of this paper, a dramatic rise in cases is observed in both Italy and the US. Supplementary Figure S4 shows this increment and how a general similarity between smell and taste trends and number of new cases in Italy and the US seems to be depicted. Despite this, the rise in new cases is accompanied by only a modest rise in popularity of searches for taste loss and smell loss, suggesting how these trends cannot be used as predictors of cases. Crucial differences that enforce this statement are pointed out in purple text in the figure. The correlation values calculated for taste and smell with the number of new cases, for the full period till October 30th, are 0.55 and 0.51 ($p < 0.05$) for Italy, and 0.35 and 0.16 ($p < 0.05$) for the US, the p-values of each estimate of the correlation were adjusted using a false discovery rate

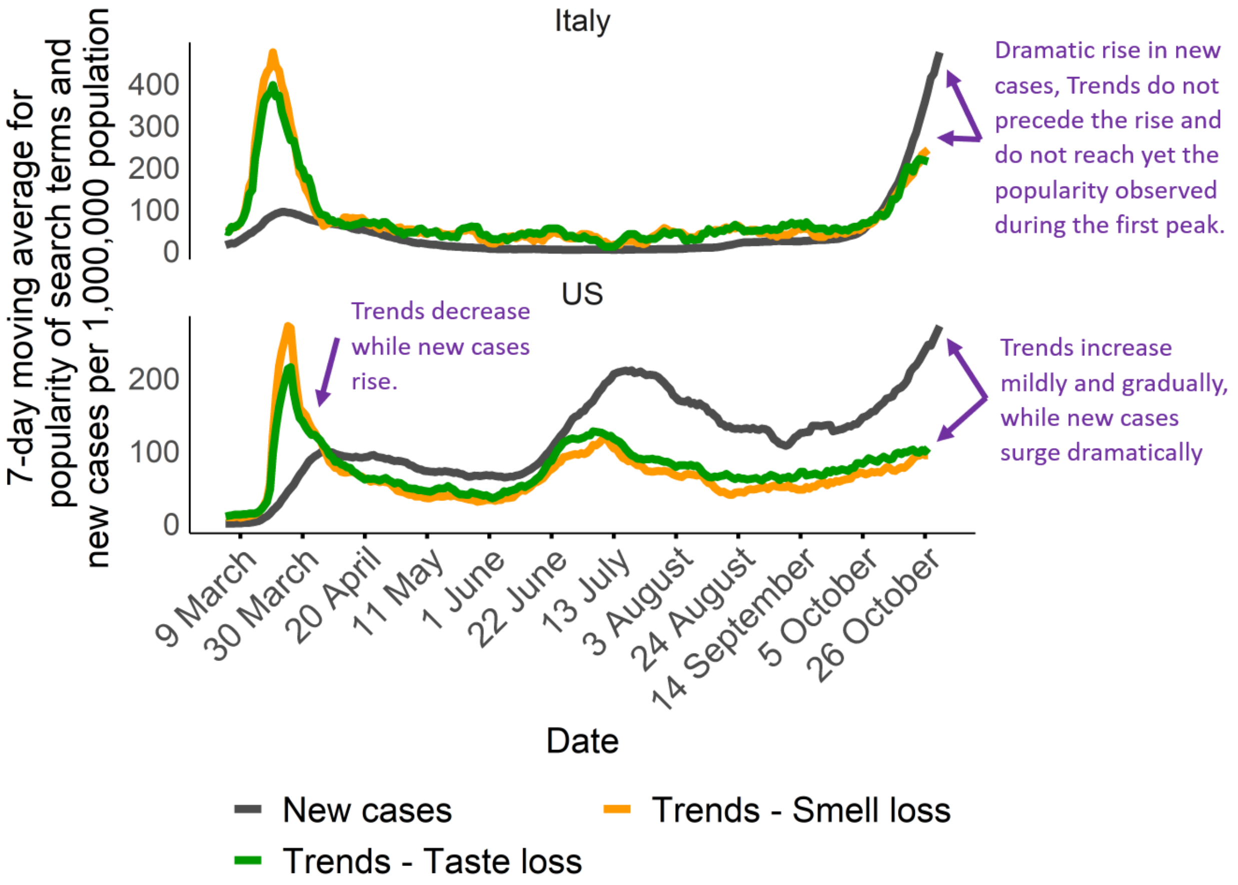

Figure S4: Extended timeline. Comparison between Google searches volume for taste loss and smell loss queries and the number of new COVID-19 cases. Showing a 7-day moving average from March 4th to October 30th for Italy and the US. The number of new cases is relative to a population of 1,000,000 in the respective country. The Google search results were normalized such that the day with the highest popularity was assigned with a value that matches the maximum number of new cases per 1,000,000 population, with the rest of the values scaled relative to that day.
